# Ways of HIV transmission in China: The effect of age, period, and cohort

**DOI:** 10.3389/fpubh.2022.941941

**Published:** 2022-09-08

**Authors:** Tang Wang, Yaohua Gu, Li Ran, Xiaodong Tan, Shuzhen Peng

**Affiliations:** ^1^School of Public Health, Wuhan University, Wuhan, China; ^2^School of Nursing, Wuhan University, Wuhan, China; ^3^Department of Health Management, Renmin Hospital in Huangpi District, Wuhan, China

**Keywords:** HIV/AID, age-period-cohort, behavior, transmission, policy

## Abstract

**Background:**

Acquired immunodeficiency syndrome (AIDS) is a global pandemic caused by human immunodeficiency virus (HIV), which is transmitted through human behaviors, such as sexual intercourse, intravenous drug injection, and blood transfusion. Rare studies have focused on the evaluation of the effects of culture, society, and HIV-related policies in adjusting people's HIV-related behaviors, i.e., ways of HIV transmission.

**Methods:**

By taking the new HIV infections in Hubei Province each year from 1995 to 2020 as the sample, our study used the Hierarchical Age-Period-Cohort (HAPC) model to analyze the effects of age, period, and cohort on the trends of ways of HIV transmission.

**Results:**

From 1995 to 2020, the number of new HIV infections in Hubei presented a general upward trend. A total of 34,636 HIV infections were reported during this period. According to the statistics of the new HIV infections in Hubei Province between 1995 and 2020, there is a negative correlation between age (−0.099, *p* < 0.001), squared age (−0.002, *p* < 0.001), and the rate of blood transmission. While there is a positive correlation between age (0.143, *p* < 0.001), squared age (0.002, *p* < 0.001), and the rate of HIV infection through sexual transmission. The significant period and cohort effects on ways of HIV transmission were also observed in the Chinese population.

**Conclusion:**

Sexual and blood transmission are the two main ways of HIV infection in China and Hubei. The trend of blood transmission is in accordance with the wave of blood trade in the early 1990s in China. The trend of sexual transmission indicates an increasing need to promote safer sexual behavior among the older population and later generations and design more tailored, innovative, and diverse HIV prevention strategies, especially for the high-risk groups.

## Introduction

Since the human immunodeficiency virus (HIV) emerged in the 1980s, an acquired immunodeficiency syndrome (AIDS) has become a global pandemic (1). As of 2020, 37.7 million people worldwide, 1.045 million people in China and 23.893 thousand people in Hubei Province were living with HIV (2, 3).

Although antiretroviral therapy (ART) has effectively blocked HIV replication in people living with HIV for decades, there is no cure for this disease, and even lifelong treatment is required (4). Thus, the chronic condition of HIV/AIDS may lead to social problems in the long run. Since 2002, the Chinese government has been implementing and promoting “the free ART regimen” strategy to improve the coverage of ART and HIV testing and adopted the “Four Frees and One Care” policy in 2004 (5). In 2003, Hubei Province implemented free ART policy, and the number of people adhering to routine ART was increased from 13 thousand in 2004 to 9.78 million in 2020. However, in China and Hubei Province, both the prevalence and the total number of people living with HIV/AIDS keep increasing (6). Due to the stigma of living with HIV/AIDS and the hidden nature of HIV transmission, it is hard to reach and control the disseminator and protect the vulnerable population. Against this backdrop, social context, such as culture, law, and HIV-related policies, plays a critical role in preventing HIV transmission.

On the other hand, social context has a significant influence on the ways of HIV transmission, as HIV is transmitted through human behaviors, such as sexual intercourse and intravenous drug injection, which are significantly influenced by local socio-cultural and political contexts (7). In China, the southwestern border area has the highest HIV/AIDS prevalence and is thus considered to be the epicenter of HIV in China (8). The spatial difference in HIV prevalence might be related to local economic development, culture, and population mobility. In addition, the deficient medical resource and personnel, poor education, and weak awareness of HIV infection contribute to the severe HIV epidemic (6). The high-risk population, such as sex workers, drug abusers, and people with risky sexual behavior, is present in a wide range of different contexts (9). Social, economic, legal, educational, and cultural contexts combine to influence local people's social networks, sexual behavior, drug abuse, etc. Although medical treatment and prevention showed effectiveness for individuals in clinical trials, there is a big gap between science and policies (10).

Therefore, eliminating HIV/AIDS can not only focus on the removal of the virus but also focus on changing people's behavior to cut HIV transmission. So far, some studies have already made great efforts in researching the mechanisms and medical treatment for HIV/AIDS (11, 12), others have focused on certain population-based interventions and strategies for reducing risky behavior and promoting preventive behavior for HIV transmission (13). However, rare studies have evaluated the effects of culture, society, and HIV-related policies in adjusting people's HIV-related behavior, i.e., ways of HIV transmission. Over the past four decades, some countries have made remarkable progress in the fight against AIDS while others have seen expanding epidemics (10). Cross-national differences in social environment, such as culture and policies, might be crucial to understand the heterogeneity of HIV transmission existing among different countries.

As a social issue, variation in HIV transmission at the population level can be explained by three effects, i.e., age, period, and birth cohort effects. The age effect reflects biological condition at an individual level, and it is associated with HIV transmission, as the pattern of risky behavior, such as sexual intercourse, varies significantly among different age groups. The period effect represents variation over time periods that affect all living age groups simultaneously, often resulting from shifts in social, cultural, economic, or physical environments (14). The period effect might work on the ways of HIV transmission *via* HIV-related policies, globalization, and the progression of medical techniques. The cohort effect is associated with changes among groups of individuals who experience an initial event, such as birth or marriage, within the same year or years (15). As the different values and patterns of behaviors between generations, the cohort effect is crucial in explaining ways of HIV transmission. In China, great social changes have been seen during the Cultural Revolution (1966–1976) and the Reform and Opening Up (in 1978), which might significantly shape the value of a certain generation. Previous studies have often compared ways of HIV transmission among different age groups across a certain period of time, thereby ignoring the potential effect of birth cohort or generation difference, which acts as a confounder between age effect and period effect (16).

Analyzing the new HIV infections in Hubei Province of China during the period 1995–2020, this study used Hierarchical Age-Period-Cohort (HAPC) model to explore: (1) the relationship between age and ways of HIV transmission; (2) how the ways of HIV transmission change with the socioeconomic environment; and (3) whether cohort effect exists in the changes of ways of HIV transmission. Considering the epidemiological distribution of HIV/AIDS and differential HIV-related policies across different provinces in China, we selected the data at the province level.

## Materials and methods

### Data sources

Hubei Province, located in central China, is among the first to report the AIDS epidemic. This study was conducted in Hubei Province, based on the data during the period 1995–2020 obtained from AIDS Prevention and Treatment Information System authorized by China Center for Disease Control.

### Variables and coding

For the newly reported HIV infection, information on demographic characteristics and the ways of transmission was collected. In view of the impacts of gender, ethnicity, education, and marital status on the ways of HIV transmission, this study took demographic characteristics, such as age, age-squared, gender, ethnicity, education, and marital status, as independent variables at the individual level. In addition, period and birth cohort were selected as independent variables at the contextual level. Age was defined as the physical age when the participants were diagnosed with HIV infection for the first time. Age was divided into 11 equal-width groups from 16–20 to 66–70 in year. The period was defined as the time (year) when the participants were diagnosed with HIV infection. The period was divided into 1 period of 2 years (1994–1995) and 5 periods of 5 years (1996–2000, 2001–2005, 2006–2010, 2011–2015, and 2016–2020). The birth cohort was defined as the actual birth year that included one 23-year birth cohort (1918–1944), eleven 5-year birth cohorts (1945–1949, 1950–1954, 1955–1959, 1960–1964, 1965–1969, 1970–1974, 1975–1979, 1980–1988, 1985–1989, 1990–1994, and 1995–1999), and one 20-year birth cohort (2000–2019). The dependent variables were the rate of transmission *via* blood and the rate of transmission *via* sexual intercourse.

### Hierarchical age period cohort

Hierarchical Age Period Cohort-Cross Classified Random Effects Model (HAPC-CCREM) was used to analyze the age-period-cohort effects on the ways of HIV transmission. The HAPC-CCREM was first proposed by Yang and Land (17) to analyze repeated cross-sectional data and combine the macro and micro-data. Moreover, the HAPC-CCREM could solve the identification conundrum among age, period, and cohort by treating period and cohort effects as macroscopic level variables (14, 18). As there is an exact linear dependency among age, period, and cohort, they cannot be estimated simultaneously. In HAPC-CCREM, variables at the individual level, such as age and other demographic characteristics, were treated as fixed effects and added into a fixed effects model. Variables at the contextual level, such as period and cohort effects, were put in a random effects model (18, 19).

All statistical analyses were performed using SAS 9.4 and *p*-values <0.05 were considered statistically significant.

## Results

### Descriptive analysis

A total of 34,636 HIV infections were reported from 1995 to 2020. Due to the omission of the period or the birth cohort data, 55 samples were excluded, resulting in a final sample of 34,581 for HAPC-CCREM. Table 1 and Supplementary Table S1 describe the demographic information and period-cohort-specific frequency of blood and sexual transmission.

**Table 1:** Encodings, means, and notes of variables.

**Variables**	**Category**	**Frequency**	**Percentage (%)**	**Note**
**Gender**	Male	26,974	77.9	
	Female	7,662	22.1	
**Age**				Mean = 49.29
				Standard Deviation = 15.46
**Ethnics**	Han-nationality	32,889	95.0	
	Others	1,747	5.0	
**Education**	Illiteracy	1,416	4.1	
	Primary school	7,695	22.2	
	Middle school	12,019	34.7	
	High school	6,779	19.6	
	University	6,483	18.7	
**Marital status**	being married	14,200	41.2	
	Single/divorced/widowed		58.8	
**Period**	1995-2020			6 periods
**Cohort**				13 cohort groups

### Age, period, and cohort effects of blood transmission

**Figure 1:**
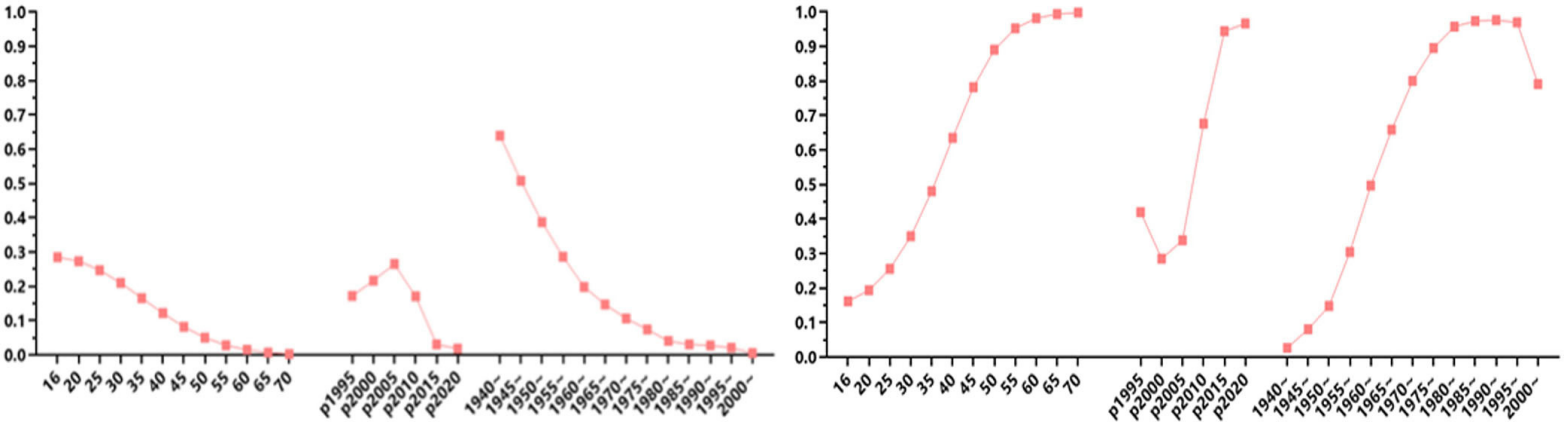
Estimation of age-period-cohort effect on the trends of human immunodeficiency virus (HIV) infection *via* blood transmission (left) and *via* sexual intercourse (right) during 1995–2020 in Hubei, China.

After controlling for all other variables, such as gender, ethnicity, marital status, and education, we obtained the net age, period, and cohort effects of blood transmission. As demonstrated in Table 2, both age (−0.099, *p* < 0.001) and squared age (−0.002, *p* < 0.001) pose negative effects, indicating that age impacts blood transmission in a non–linear way. During 1995–2020, the rates of blood transmission were decreased with age and reached the lowest point among people aged 70 years and over (Figure 1). For period effect, the rates of blood transmission were increased to the highest point in the period 2001–2005 (*p* < 0.05) and then sharply declined from 2005 to 2020 (*p* < 0.001; Figure 1 and Supplementary Table S2). Regarding the cohort effect, the rates of blood transmission showed a protracted downward trend from 1940 to 1959 (*p* < 0.05) and 1980 to 2020 (*p* < 0.05).

**Table 2:** Estimation of age-period-cohort effect on the trends of human immunodeficiency virus (HIV) infection *via* blood transmission during 1995–2020 in Hubei, China.

	**Coefficient**	**Standard deviation**	***P*-value**
**Fixed effects**	
Intercept	−0.807	0.556	0.206
Age	−0.099	0.009	<0.001
Age^2^	−0.002	0.000	<0.001
Gender	0.089	0.061	0.172
Ethnic	−0.560	0.127	0.014
Marital	−0.016	0.197	0.903
Education	−0.451	0.102	0.003
**Random effects**	
Time period	1.087	0.707	0.062
Birth cohort	1.379	0.893	0.061
**Goodness of fit**	
−2 Log Likelihood	222060.900		

For demographic factors, both ethnicity and education influenced HIV transmission *via* blood significantly (*p* < 0.05): the rate of blood transmission is lower among Han Chinese and those who are highly educated (Table 2).

### Age, period, and cohort effects of sexual transmission

After controlling for all other variables, such as gender, ethnicity, marital status, and education, we obtained the net age, period, and cohort effects of sexual transmission. As demonstrated in Table 3, both age (0.143, *p* < 0.001) and squared age (0.002, *p* < 0.001) have positive effects, indicating that age impact sexual transmission in a nonlinear way. During 1995–2020, the rates of sexual transmission had increased with age and reached a peak among people aged 70 or over (Figure 1). For period effect, the rates of sexual transmission had decreased from 1995 to 2000 (*p* < 0.05) and then increased rapidly from 2005 thereafter, and the increases in 2015 and 2020 were significant (*p* < 0.05). The period effect generally shows an “S-shaped” pattern as shown in Figure 1 and Supplementary Table S3. Regarding cohort effect, the rates of sexual transmission were increased steadily from birth cohort 1940 to 1990 and then decreased from birth cohort 1990 to 2000. The increases in birth cohorts 1940, 1945, 1950, 1955, 1980, 1985, and 1990 were significant (*p* < 0.05), and the decreases in 1995 were significant (0.002, *p* < 0.001), as shown in Figure 1 and Supplementary Table S3.

**Table 3:** Estimation of age-period-cohort effect on the trends of human immunodeficiency virus (HIV) infection *via* sexual transmission during 1995–2020 in Hubei, China.

	**Coefficient**	**Standard deviation**	***P*-value**
**Fixed effects**	
Intercept	−1.429	0.856	0.156
Age	0.143	0.012	<0.001
Age^2^	0.002	0.000	<0.001
Gender	0.178	0.013	<0.0001
Ethnic	0.003	0.000	<0.0001
Marital	−0.065	0.117	0.589
Education	0.747	0.227	0.006
**Random effects**	
Time period	2.247	1.485	0.065
Birth cohort	3.932	1.968	0.023
**Goodness of fit**	
−2 Log likelihood	244,615.200		

For demographic factors, ethnicity, gender, and education all influenced sexual transmission remarkably (*p* < 0.05): the rate of sexual transmission is higher among the men, Han Chinese, and better-educated groups (Table 3).

## Discussion

Based on the data of new HIV infections in the Hubei Province of China during 1995**–**2020, this article analyzed the age, period, and birth cohort variations in the ways of HIV transmission, especially for sexual and blood transmission through HAPC-CCREM. Two main findings can be observed. First, there were significant age, period, and cohort effects of HIV transmission among the Chinese population. There was a curvilinear association between age and HIV transmission *via* blood and sexual intercourse. The age, period, and cohort effects of blood transmission showed a downward trend while these effects of sexual transmission showed an upward trend. Second, sociodemographic factors, such as education, ethnicity, and gender, could remarkably influence how ways of HIV transmission change with time. This is in line with current evidence in other countries where policies posed an impact on new HIV infection (13).

When using the APC model to understand changes with time (20), the age effect refers to biological changes and changes due to accumulation of social experience or transition of social role. The period effect refers to social, cultural, or environmental changes occurring with time and impacting the whole population from whichever age group. The cohort effect refers to the mixed effects of age and period (6). In addition, the period effect focused on the change of social contexts (21), such as the updates of HIV-related policies. Regarding the variation in AIDS transmission, distribution, and related policies between the provinces, we selected province-level data to conduct this research.

Hubei Province locates in central China with its gross domestic product (GDP) ranking seventh out of 31 province-level districts (22). A total of 23,893 people are living with HIV/AIDS in Hubei Province, ranking 12th in China. Since the first HIV case was reported in 1988 in Hubei, HIV has spread fast in a hidden, undetectable, and untreatable way. In response to the HIV pandemic, Hubei Province announced a series of policies to control its transmission.

In our study, we mainly analyzed HIV transmission *via* sexual intercourse and blood, which are the two main ways of HIV transmission in China and Hubei. According to our results, the proportion of blood transmission varied significantly among different age groups. For older age groups, the rate of HIV/AIDS transmission *via* blood transmission is relatively lower. This finding is in accordance with the facts in the wave of blood merchants in the early 1990s in China (23) when commercial blood donation was encouraged and even considered as the main shortcut for farmers to change their lives. At that time, younger donors were more popular in the blood trade, while old donors especially those over 50 were often rejected. In the 1990s, lack of awareness and sufficient knowledge about HIV, blood merchants in certain areas near Hubei Province were overwhelmed, leading to a dramatic increase in HIV transmission *via* blood. In 1996 and 1997, China proposed “regulations on the administration of blood products” and “blood donation law in the People's Republic of China” aiming to control HIV transmission by forbidding the blood trade. Thereafter, blood could not be used to trade for money, and the blood donors were routinely tested for HIV antibodies. We find that the rates of blood transmission peaked in the period 2001–2005 from 1995 to 2000. The two HIV-related policies enacted in 1996 and 1997, respectively, appear to be ineffective as the rate of blood transmission kept rising. This might be explained by two reasons: firstly, the intention and ability of HIV testing before 2000 were low. The number of new HIV infections was obviously lower than the actual number of HIV infections. Secondly, the illegal blood trade still existed for years after the introduction of two HIV policies. Against the backdrop of China's immature economy and labor market, blood trade was important for those with poor socioeconomic condition to earn their living. In 2007, 22,000 out of 75,000 people living with HIV/AIDS in China were infected through blood trade or transfusion (23). Our findings suggest that the two HIV-related policies took effect in 2005 and since then the rates of blood transmission started declining.

For the rates of sexual transmission, age, period, and cohort effects significantly explained its variation. The rates of sexual transmission were significantly higher in the older group, which is similar to the findings from other countries where the prevalence of HIV infections increased fastest among people aged 50–80 years (24). Opposite to the common assumption that the older population has rare or no need for sexual activity, as people live longer, older individuals continue to enjoy sexual activity with multiple reasons for showing risky sexual behavior (25). Although marital status does not have a significant influence on the rates of sexual transmission, gender does. Men living with HIV/AIDS presented a higher rate of sexual transmission, especially for those who were older. As for the birth cohort effect, the rate of sexual transmission was increased steadily among people born between 1940 and 1994 and decreased suddenly for people born after 1995. This might be explained by the intragenerational differences in sexual concepts or culture. According to the socialization theory and life course theory, people who went through similar experiences and periods of society process tend to hold similar values (26). The higher rates of sexual transmission among the late birth cohort may indicate more active or risky sexual behavior. The decrease observed in the birth cohort after 1995 might be explained by two reasons: firstly, people in this birth cohort were at their beginning of sexual experience; secondly, the treatment for HIV/AIDS, such as ART, might play an impact on blocking HIV transmission. There are several limitations in our study. First, while the HAPC model could demonstrate the pure influence of age, period, and cohort effect on HIV transmission, it is unable to explain the underlying mechanisms of the variances. Second, we were unable to understand how occupation might influence HIV transmission owing to inadequate data on occupation.

Our increasing knowledge on the ways of HIV transmission is shaping the development of new, more sophisticated intervention strategies (27). To the best of our knowledge, this is the first study to analyze the age, period, and cohort effect in impacting ways of HIV transmission. We analyzed province-level data in China aiming to figure out how social context might impact on the ways of HIV transmission. Our findings might throw light on the direction and intervention to block the spread of sexual transmission of HIV.

## Data availability statement

The raw data supporting the conclusions of this article will be made available by the authors, without undue reservation.

## Ethics statement

The studies involving human participants were reviewed and approved by Wuhan University College of Medicine. The patients/participants provided their written informed consent to participate in this study.

## Author contributions

TW: writing—original draft and data analysis. YG and LR: data analysis, reviewing, and editing. YG: data analysis and revised the manuscript. SP: revised the manuscript. XT and SP: conception, reviewing, and editing. All authors contributed to the article and approved the submitted version.

## Conflict of interest

The authors declare that the research was conducted in the absence of any commercial or financial relationships that could be construed as a potential conflict of interest.

## Publisher's note

All claims expressed in this article are solely those of the authors and do not necessarily represent those of their affiliated organizations, or those of the publisher, the editors and the reviewers. Any product that may be evaluated in this article, or claim that may be made by its manufacturer, is not guaranteed or endorsed by the publisher.
