# Supplementary material for: Ways of HIV transmission in China: The effect of age, period, and cohort
Source: Front Public Health. 2022 Sep 8;10:941941. doi: 10.3389/fpubh.2022.941941 (PMC9493025; doi:10.3389/fpubh.2022.941941)
Supplement: Supplementary file 1 [file Data_Sheet_1.PDF]

Supplementary Material

1 Supplementary Tables

1.1 Supplementary Table 1 Period-cohort-specific case numbers and frequency of blood and sex transmission (1995-2020)

Cohort	Period																				
	-1995			1996-2000			2001-2005			2006-2010			2011-2015			2016-2020			Total		
	Case	Blood	Sex	Case	Blood	Sex	Case	Blood	Sex	Case	Blood	Sex	Case	Blood	Sex	Case	Blood	Sex	Case	Blood	Sex
-1940	0	0	0	4	2	2	56	35	11	110	16	71	420	1	418	628	1	627	1218	55	1129
1945-	0	0	0	7	7	0	76	58	14	107	26	71	501	6	494	753	1	751	1444	98	1330
1950-	4	3	1	11	11	0	200	164	21	215	58	136	552	13	539	1062	0	1060	2044	249	1757
1955-	4	4	0	18	18	0	230	189	32	245	75	159	702	11	689	1415	10	1402	2614	307	2282
1960-	5	4	0	30	25	3	359	289	59	375	137	220	981	25	955	1740	10	1729	3490	490	2966
1965-	7	7	0	21	20	1	471	362	92	535	194	318	1116	38	1077	1877	24	1853	4027	645	3341
1970-	3	2	1	15	13	2	425	311	94	530	174	336	1309	55	1255	1835	24	1808	4117	579	3496
1975-	1	1	0	14	12	2	190	116	65	447	112	316	962	34	928	1351	40	1308	2965	315	2619
1980-	0	0	0	5	3	2	70	33	34	361	50	298	1110	39	1071	1396	19	1372	2942	144	2777
1985-	0	0	0	0	0	0	34	21	12	329	40	281	1479	24	1453	1955	36	1917	3797	121	3663
1990-	0	0	0	0	0	0	11	8	0	79	27	50	1185	17	1168	2246	21	2221	3521	73	3439
1995-	0	0	0	3	0	0	32	14	0	18	4	1	245	6	234	1686	8	1676	1984	32	1911
2000-	0	0	0	0	0	0	16	1	0	37	0	0	32	1	0	333	0	303	418	2	303
Total	24	21	2	128	111	12	2170	1601	434	3388	913	2257	10594	270	10281	18277	194	18027	34581	3110	31013

1.2 Supplementary Table 2. Estimation results of HAPC model for HIV infection via blood

Period	Coefficient	Standard Error	*P*-value	Cohort	Coefficient	Standard Error	*P*-value
1995	0.173	0.547	0.293	1940	0.640	0.560	<0.001
2000	0.217	0.520	0.099	1945	0.509	0.529	<0.001
2005	0.266	0.510	0.027	1950	0.388	0.509	0.001
2010	0.171	0.510	0.269	1955	0.287	0.499	0.014
2015	0.030	0.516	0.011	1960	0.199	0.489	0.126
2020	0.019	0.522	0.001	1965	0.148	0.484	0.423
				1970	0.107	0.483	0.972
				1975	0.075	0.486	0.437
				1980	0.041	0.495	0.041
				1985	0.030	0.506	0.009
				1990	0.028	0.522	0.007
				1995	0.021	0.545	0.002
				2000	0.005	0.719	<0.001

1.3 Supplementary Table 3. Estimation results of HAPC model for HIV infection via sexual transmission

Period	Coefficient	Standard Error	*P*-value	Cohort	Coefficient	Standard Error	*P*-value
1995	0.420	0.947	0.223	1940	0.027	0.779	<0.001
2000	0.286	0.814	0.032	1945	0.081	0.746	<0.001
2005	0.339	0.774	0.052	1950	0.148	0.719	0.000
2010	0.676	0.773	0.901	1955	0.305	0.704	0.019
2015	0.944	0.777	0.010	1960	0.497	0.692	0.223
2020	0.966	0.783	0.001	1965	0.659	0.686	0.800
				1970	0.800	0.683	0.419
				1975	0.896	0.686	0.055
				1980	0.957	0.694	0.001
				1985	0.973	0.705	<0.001
				1990	0.976	0.722	<0.001
				1995	0.970	0.739	0.000
				2000	0.791	0.757	0.510
